# Ecosystem Carbon Storage in Alpine Grassland on the Qinghai Plateau

**DOI:** 10.1371/journal.pone.0160420

**Published:** 2016-08-05

**Authors:** Shuli Liu, Fawei Zhang, Yangong Du, Xiaowei Guo, Li Lin, Yikang Li, Qian Li, Guangmin Cao

**Affiliations:** 1 Northwest Institute of Plateau Biology, Chinese Academy of Sciences, Xining, Qinghai, China; 2 University of Chinese Academy of Sciences, Beijing, China; Peking University, CHINA

## Abstract

The alpine grassland ecosystem can sequester a large quantity of carbon, yet its significance remains controversial owing to large uncertainties in the relative contributions of climate factors and grazing intensity. In this study we surveyed 115 sites to measure ecosystem carbon storage (both biomass and soil) in alpine grassland over the Qinghai Plateau during the peak growing season in 2011 and 2012. Our results revealed three key findings. (1) Total biomass carbon density ranged from 0.04 for alpine steppe to 2.80 kg C m^-2^ for alpine meadow. Median soil organic carbon (SOC) density was estimated to be 16.43 kg C m^-2^ in alpine grassland. Total ecosystem carbon density varied across sites and grassland types, from 1.95 to 28.56 kg C m^-2^. (2) Based on the median estimate, the total carbon storage of alpine grassland on the Qinghai Plateau was 5.14 Pg, of which 94% (4.85 Pg) was soil organic carbon. (3) Overall, we found that ecosystem carbon density was affected by both climate and grazing, but to different extents. Temperature and precipitation interaction significantly affected AGB carbon density in winter pasture, BGB carbon density in alpine meadow, and SOC density in alpine steppe. On the other hand, grazing intensity affected AGB carbon density in summer pasture, SOC density in alpine meadow and ecosystem carbon density in alpine grassland. Our results indicate that grazing intensity was the primary contributing factor controlling carbon storage at the sites tested and should be the primary consideration when accurately estimating the carbon storage in alpine grassland.

## Introduction

China’s terrestrial ecosystems (both vegetation and soils) have been estimated to have sequestered 20.8–26.8% of the carbon released in industrial CO_2_ emissions during 1981–2000 [1]. It is reported that the grasslands of China (331×10^6^ ha) cover only 6–8% of the total world grassland area but contain 9–16% of the world’s total carbon [2]. To date, a large number of estimates of the forest ecosystem carbon stocks in China have been reported [3,4], but a comprehensive assessment of carbon storage in China’s grasslands is still lacking [5]. Estimating the level of carbon stored in living vegetation and soil organic matter in grassland ecosystems has been limited by the lack of direct measurements and the large spatial heterogeneity of grassland ecosystems [6–8]. Accurately estimating carbon storage in vegetation and soil is not only essential for understanding current levels of carbon pools, but also for mapping how these terrestrial ecosystem carbon pools change over time, which is critical for evaluating the global carbon budget, the main predictor of climate change [9,10].

Alpine grasslands on the Tibetan Plateau constitute 34.3% of the total grassland in China [11] and comprise 56.4% of the total grasslands biomass carbon in China [6]. This indicates that carbon storage in alpine grasslands likely plays a significant role in global carbon cycles [12]. Surprisingly, the importance of carbon storage, in particular biomass carbon, in alpine grassland has not been well recognized due to the variations in topography and access [13]. Biomass data are needed to estimate levels of carbon storage, and are best provided by the collection of field data [4,14]. Currently there are very limited field observations of biomass in the alpine grassland of the Qinghai Plateau, so to accurately estimate ecosystem (biomass and soil) carbon storage, ground-based observational data are required.

Both biomass and soil carbon stock in terrestrial ecosystems may respond to climate change and disturbance caused by human activities [15,16]. Alpine ecosystems have been shown to have a greater and more rapid response to warming scenarios [17], and are also influenced by human activities such as livestock grazing [18]. To accurately estimate ecosystem carbon budget, the effects of climate factors and grazing intensity must be taken into account.

The goals of this study are: (1) to estimate total ecosystem (both biomass and soil) carbon storage in alpine grassland, and (2) to quantitatively define the influences of climate and grazing on the carbon density in alpine grassland. This study will help to clarify the current levels of carbon storage in alpine grassland, and will enhance our understanding of the roles that climate change and grazing intensity play on carbon budget.

## Materials and Methods

### Study area

**Fig 1:**
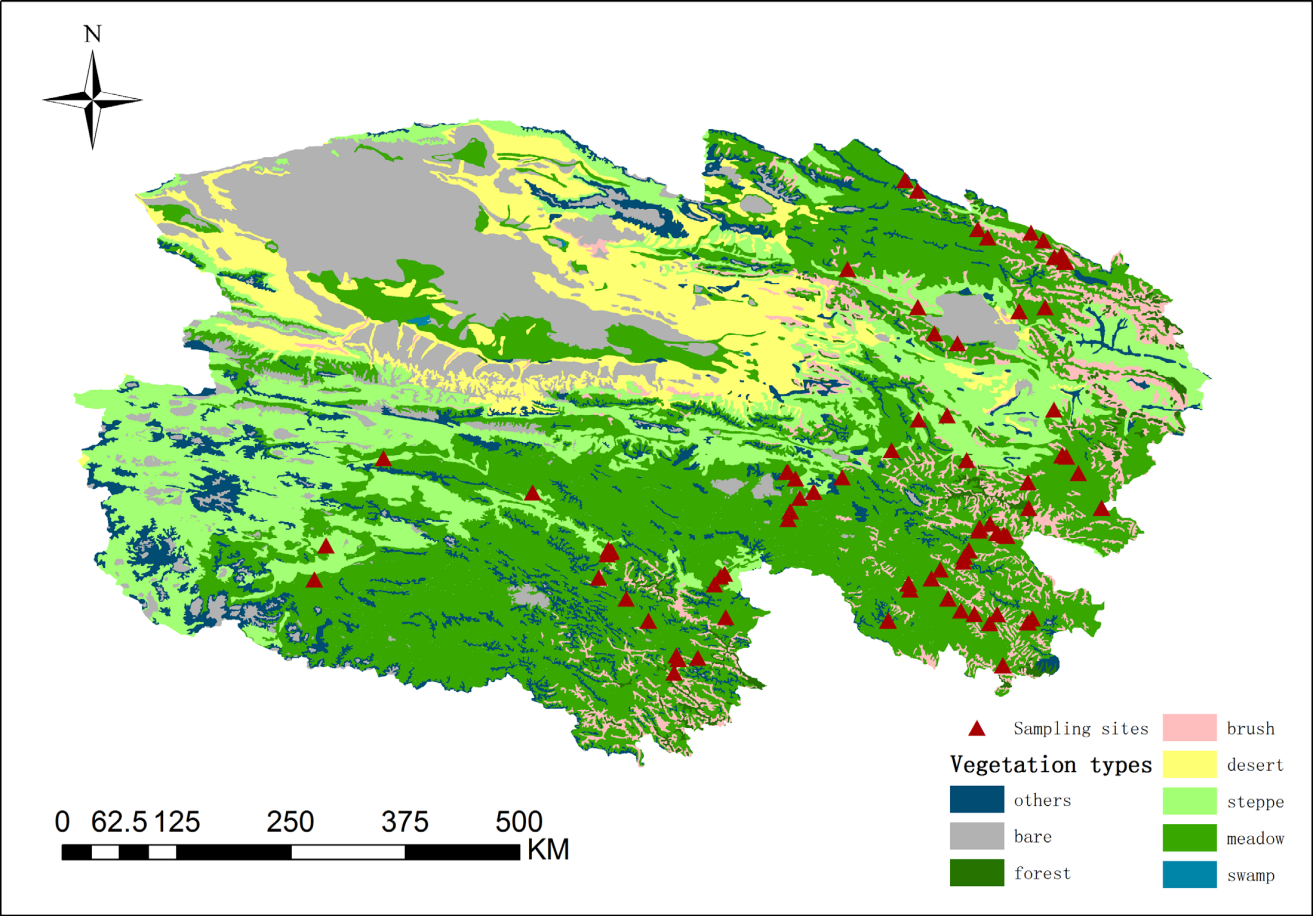
Spatial distribution of sampling sites in alpine grasslands on the Qinghai Plateau. The vegetation map of the plateau was obtained from China’s vegetation atlas at a scale of 1: 1,000,000 (Chinese Academy of Sciences, 2001). Also shown are locations of the 115 sites surveyed during 2011–2012.

The study sites were located on the Qinghai-Tibetan Plateau in Qinghai Province. The area covered 36.37×10^4^ km^2^, extending from 92.17 to 101.75°E in longitude and from 30.29 to 38.60°N in latitude [19]. The region experiences a typical plateau climate, with a cool, short summer and a cold, long and dry winter, and thus the growing season is short compared to other areas at similar latitudes [17]. The average elevation was 4000 m, the mean annual temperature ranged from -5°C to 12°C [20], and the annual precipitation was 500 mm—800 mm, of which over 80% fell during the summer season [21]. Alpine grassland was the main vegetation type in the area. Based on China’s vegetation classification system, we divided the alpine grasslands in this region into two types: alpine steppe and alpine meadow [22]. The distribution of grassland types was obtained from China’s vegetation atlas at a scale of 1:1,000,000 (Chinese Academy of Sciences, 2001). This classification, along with the locations of the 115 sampling sites, is shown in Fig 1. The area of the alpine steppe and alpine meadow was extracted from the map.

### Sampling method

We aimed to measure the biomass of above- and below-ground carbon as well as soil carbon at 115 sites during 2011 and 2012. At each site, no specific permits were demanded for collecting samples and the field studies did not involve endangered or protected species. The sites included mostly winter pasture (grazing from November to April) and some summer pasture (grazing from May to October). At each site (10 m × 100 m), five 0.25 m^2^ (0.5 m × 0.5 m) and five 1 m^2^ (1 m × 1 m) quadrats were sampled in the alpine meadow and alpine steppe, respectively. The geographical location of each site was determined by a Global Positioning Satellite (GPS) device. At each site the altitude, longitude, latitude, grassland types, total vegetation cover, dominant species and land use types were recorded.

Peak above-ground biomass (AGB) was measured by the clipping method in both winter and summer pasture during July and September. Quadrats were located at 20 m intervals along the 100 m × 10 m sites and all plants (both alive and dead) in the five small quadrats (0.5 m × 0.5 m for alpine meadow and 1 m × 1 m for alpine steppe) were harvested to ground level. Below-ground biomass (BGB) was also measured during the growing season, following AGB collection in the quadrats. To assess BGB, a soil core (6 cm diameter) was used to collect samples and was divided into seven depth increments (0–5, 5–10, 10–20, 20–30, 30–50, 50–70 and 70–100 cm). Five soil samples from each depth interval on the same quadrats were lumped together and then cleaned under running water to remove soil particles. Roots were passed through a 2-mm sieve to remove fine roots (< 2 mm). Live and dead roots could not be distinguished; hence, the BGB values included both live and dead roots [23]. The biomass samples (both AGB and BGB) were oven dried at 65°C to a constant mass and then weighed to the nearest 0.1 g. The carbon concentration of biomass (both live and dead biomass) was measured by the dry combustion method using an elemental analyzer (2400 II CHNS/O, Perkin-Elmer, USA). The biomass carbon density of each site was calculated by multiplying the carbon concentration and the respective mass of each AGB and BGB component within the site [24].

Soil samples were collected using the same method as that for BGB samples and were divided into seven depth increments (0–5, 5–10, 10–20, 20–30, 30–50, 50–70 and 70–100 cm). In the laboratory, the soil samples were air-dried and then sieved to 0.25 mm. Soil carbon concentration was also measured by elemental analyzer (2400 II CHNS/O, Perkin-Elmer, USA). We calculated SOC density in the top 1 m of each sample using the method of stratified cumulative sum (Eq 1).

$$SOCD=\sum_{i=1}^{n}SOC_{i}\times P_{i}\times D_{i}\times (1-C_{i})/100$$ (1)

Where *SOCD* is the SOC density (kg C m^-2^) of the profile, *SOC*_*i*_ is the SOC concentration (g kg^-1^), *Ρ*_*i*_ is the bulk density (g cm^-3^), *D*_*i*_ is the soil thickness (cm), and *C*_*i*_ is the volume percent of gravel (particle sizes > 2 mm) in layer *i*, respectively.

The mean annual temperature (MAT) and mean annual precipitation (MAP) were extracted from the climate database of Qinghai Province for the period 1971–2010 [25]. Livestock data for each county were obtained from the prairie station of Qinghai Province during 2011–2012 and calculated according to standard conversions in which one yak is equivalent to 4.5 sheep units and one horse is equivalent to 6 sheep units (Agricultural industry standard of the people's Republic of China, 2002).

### Statistical analysis

For all data analysis, preliminary normality testing was carried out to ensure the normality of the data. Linear mixed-effects model (LME) analysis was performed to test the possible dependency of ecosystem carbon density on environmental factors. Then ordinary least squares (OLS) regression analysis was conducted to evaluate the relationships between ecosystem carbon density and the climate or grazing factors. We have excluded the effects of the other explanatory variables while assessing the effect of one explanatory variable. All statistical analyses were performed in SPSS 16.0 (SPSS Inc. Chicago, IL, USA). In all cases, a *P* value <0.05 was considered to be significant.

## Results

### Biomass carbon in alpine grassland

Observed AGB ranged from 4.17 to 355.38 g m^-2^. Median BGB of the alpine steppe and alpine meadow are 1109.04 and 2968.73 g m^-2^, respectively. The ratio of BGB to AGB varied between sites, ranging from 0.95–52.96. For the two alpine grassland types, the median ratio of BGB to AGB was 20.40, and a higher median ratio of BGB to AGB was observed in the alpine meadow (21.38) (Table 1).

**Table 1:** Values of biomass and biomass carbon for alpine grassland on the Qinghai Plateau

Grassland types	n	AGB ^a^ (g m^-2^)			BGB ^b^ (g m^-2^)			Ratio of BGB to AGB			AGB carbon density (g C m^-2^)			BGB carbon density(g C m^-2^)		
		low	median	high	low	median	high	low	median	high	Low	median	high	low	median	high
Alpine steppe	23	4.17	61.64	191.69	89.27	1109.04	4725.97	4.38	16.16	41.82	0.98	18.89	80.59	35.55	385.50	1788.31
Alpine meadow	92	20.68	129.60	355.38	176.48	2968.73	6493.34	0.95	21.38	52.96	3.83	41.04	155.29	55.34	1185.57	2714.98
Alpine grassland	115	4.17	111.23	355.38	89.27	2361.11	6493.34	0.95	20.40	52.96	0.98	39.06	155.29	35.55	908.33	2714.98

^a^ AGB, aboveground biomass

^b^ BGB, belowground biomass.

**Fig 2:**
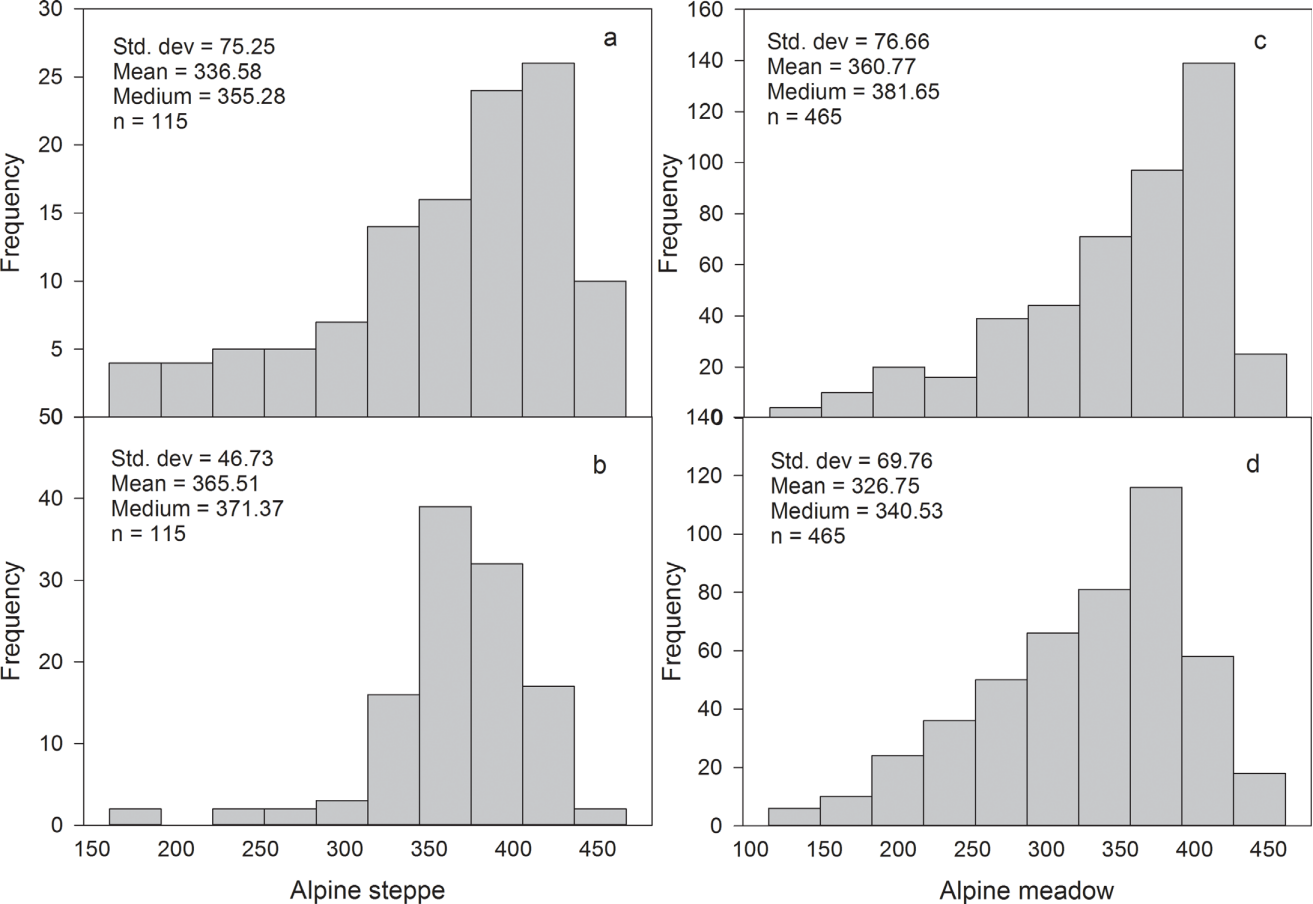
**Frequency distributions of above-ground biomass (AGB) carbon concentration and below-ground biomass (BGB) carbon concentration of alpine steppe (a and b) and alpine meadow (c and d).** The unit of the biomass carbon concentration is g kg^-2^.

The mean AGB carbon concentration differed between the alpine steppe (33.66%) and the alpine meadow (36.03%) (Fig 2). The mean BGB carbon concentrations in the alpine steppe and the alpine meadow were 36.55% and 32.44%, respectively (Fig 2). Based on our measurements, the mean carbon concentration of both AGB and BGB in alpine grassland was approximately 35%.

Both AGB and BGB carbon densities exhibited large variations across the alpine grassland, ranging from 0.98 g C m^-2^ to 155.29 g C m^-2^ for ABG carbon density and 35.55 g C m^-2^ to 2714.98 g C m^-2^ for BGB carbon density. Median AGB carbon density (41.01 g C m^-2^) and BGB carbon density (1185.57 g C m^-2^) in alpine meadow were higher compared to levels in alpine steppe (18.89 and 385.50 g C m^-2^) (Table 1).

### Ecosystem carbon storage in alpine grassland

Total biomass carbon density varied between sites, ranging from 0.04 kg C m^-2^ to 2.80 kg C m^-2^. Median values of biomass carbon density were 0.40 kg C m^-2^ and 1.19 kg C m^-2^ in alpine steppe and alpine meadow, respectively. Relatively low biomass carbon density was observed in the alpine steppe. SOC density in the top 1 m differed between sites and grassland types, ranging from 1.91 kg C m^-2^ for alpine steppe to 25.91 kg C m^-2^ for alpine meadow. Median SOC density in the top 1 m in alpine grassland was estimated to be 16.43 kg C m^-2^. Ecosystem carbon density in alpine grassland varied widely, from 1.95 kg C m^-2^ for alpine steppe to 28.71 kg C m^-2^ for alpine meadow, and the median value was estimated to be 17.40 kg C m^-2^. Higher ecosystem carbon density was observed in the alpine meadow, with both biomass and soil carbon densities within the alpine meadow being higher than those in alpine steppe (Table 2).

**Table 2:** Ecosystem (biomass and soil) carbon density in alpine grassland on the Qinghai Plateau.

Grassland types	Biomass carbon density (kg C m^-2^)			Soil organic carbon density (kg C m^-2^)			Ecosystem carbon density (kg C m^-2^)		
	Low	Median	High	Low	Median	High	Low	Median	High
Alpine steppe	0.04	0.40	1.84	1.91	9.30	21.05	1.95	9.70	22.89
Alpine meadow	0.06	1.19	2.80	6.79	17.86	25.91	6.85	19.05	28.71
Alpine grassland	0.04	0.97	2.80	1.91	16.43	25.91	1.95	17.40	28.71

The respective areas of alpine steppe and alpine meadow were 5.83×10^4^ km^2^ and 23.66×10^4^ km^2^, which accounted for 80% of the total grassland area in Qinghai Province. Ecosystem carbon storage in the alpine steppe was estimated to be 0.56 Pg, with a large majority (96%) occurring as stored carbon in the soil. However, the ecosystem carbon storage in alpine meadow reached 4.51 Pg, with 94% (4.23 Pg) being soil organic carbon and 6% (0.28 Pg) biomass carbon. The percentage of biomass carbon in alpine meadow (6%) was slightly higher than in alpine steppe (4%). The overall total carbon storage in alpine grassland was estimated to be 5.14 Pg, of which 94% (4.85 Pg) was soil organic carbon and 6% (0.29 Pg) was biomass carbon (Table 3).

**Table 3:** Ecosystem carbon storage in alpine grassland of the Qinghai Plateau

Grassland types	Area (10^4^km^2^)	Biomass carbon storage (Pg^a^ C)			Soil organic carbon storage (Pg C)			Ecosystem carbon storage (Pg C)		
		Low	Median	High	Low	Median	High	Low	Median	High
Alpine steppe	5.83	0.002	0.02	0.11	0.11	0.54	1.23	0.11	0.56	1.33
Alpine meadow	23.66	0.01	0.28	0.66	1.61	4.23	6.13	1.62	4.51	6.79
Alpine grassland	29.49	0.01	0.29	0.83	0.56	4.85	7.64	0.58	5.14	8.47

^a^1Pg = 10^15^g.

### Factors influencing ecosystem carbon density

**Fig 3:**
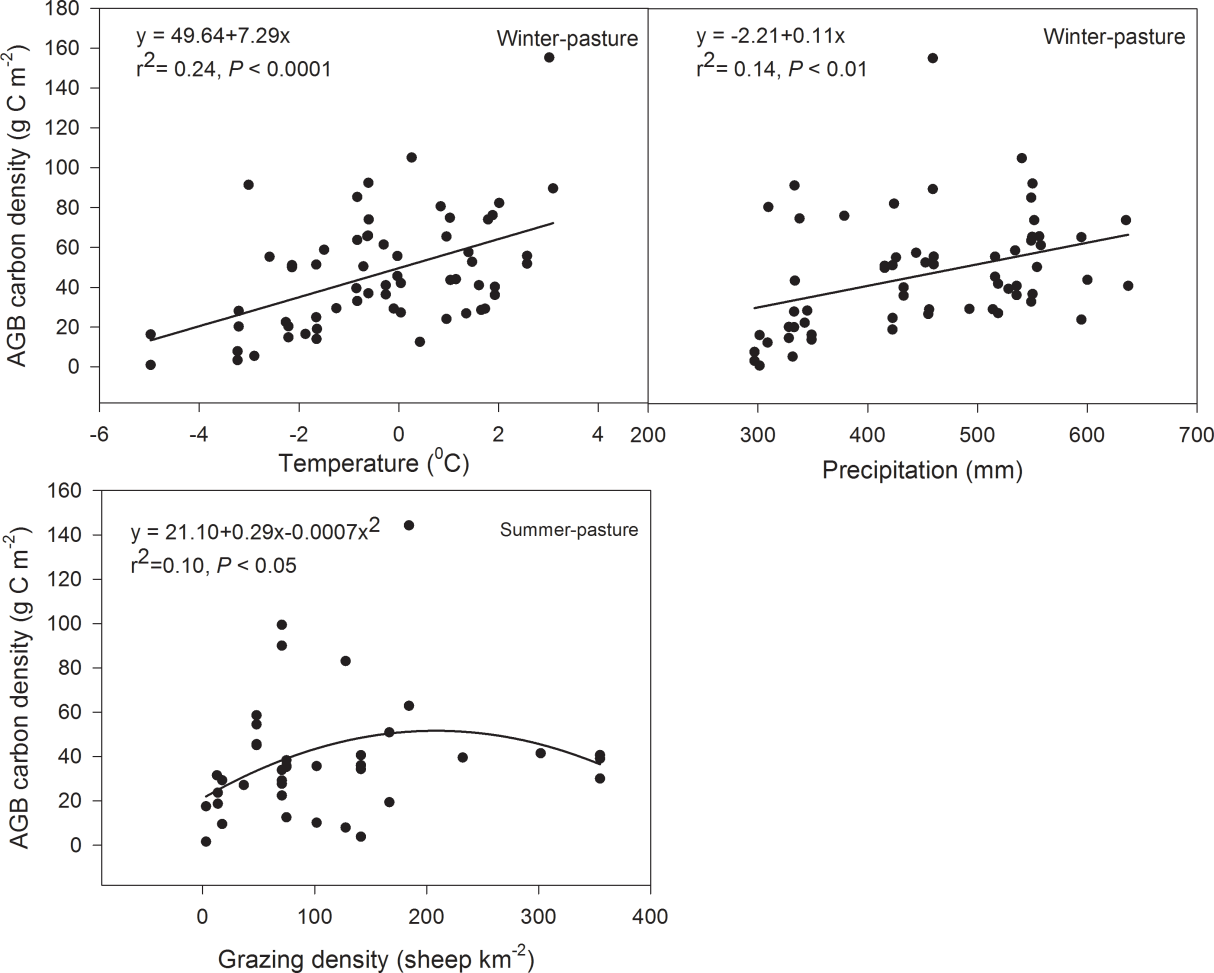
Relationships between above-ground biomass (AGB) carbon density and environmental (climate and grazing) factors in alpine grassland. Variation of annual average temperature and annual average precipitation from 1971–2010 in alpine grassland.

Grazing type alone had a great impact on AGB carbon density in the overall analysis. When the two pasture types were analyzed separately, there was a significant positive effect of temperature and precipitation on AGB carbon density in winter pasture (*F* = 4.80, *P* = 0.03), while in the summer pasture, the AGB carbon density initially increased but then decreased with increasing grazing intensity (Table 4, Fig 3). We have excluded the effects of the other explanatory variables while assessing the effect of one explanatory variable.

**Table 4:** Effects of environmental factors on biomass carbon and SOC density in a linear mixed-effects model (LME).

		Alpine grassland			Winter pasture			Summer pasture		
		^*j*^ *df*	*F*	^*k*^ *P*	*df*	*F*	*P*	*df*	*F*	*P*
AGB ^a^ carbon Density (g C m^-2^)	GT ^d^	1	1.18	0.28						
	GS ^e^	1	6.84	0.01						
	T ^f^	1	3.82	0.05	1	5.68	0.02	1	1.12	0.30
	P ^g^	1	0.75	0.39	1	0.14	0.71	1	1.76	0.20
	GD ^h^	1	0.81	0.37	1	0.00	0.98	1	3.93	0.05
	T×P ^i^	1	1.93	0.17	1	4.80	0.03	1	1.70	0.20
	A	Alpine grassland				Alpine steppe			Alpine meadow		
BGB ^b^ carbon density (g C m^-2^)	GT	1	14.70		<0.001						
	GS	1	1.84	0.18						
	T	1	5.42	0.02	1	0.85	0.37	1	4.56	0.04
	P	1	0.69	0.41	1	1.12	0.31	1	4.31	0.04
	GD	1	0.24	0.63	1	0.85	0.37	1	1.14	0.29
	T×P	1	7.34	0.01	1	1.09	0.31	1	4.62	0.04
SOC ^c^ density (kg C m^-2^)	GT	1	14.36	<0.001						
	GS	1	3.44	0.07						
	T	1	0.59	0.44	1	8.21	0.01	1	0.43	0.52
	P	1	0.11	0.73	1	0.16	0.70	1	0.13	0.72
	GD	1	12.35	<0.001	1	0.25	0.62	1	7.43	0.01
	T×P	1	0.03	0.87	1	9.04	0.01	1	0.94	0.34
Ecosystem carbon density (kg C m^-2^)	GT	1	17.11	<0.001						
	GS	1	4.37	0.04						
	T	1	1.01	0.32	1	6.00	0.03	1	0.30	0.59
	P	1	0.00	0.99	1	0.02	0.89	1	0.46	0.50
	GD	1	8.44	0.004	1	10.70	0.004	1	4.66	0.03
	T×P	1	0.16	0.69	1	5.66	0.03	1	0.71	0.40

^a^ AGB carbon density, aboveground biomass carbon density

^b^ BGB carbon density, belowground biomass

^c^ SOC density, soil organic carbon density

^d^ GT, grassland types, including alpine steppe and alpine meadow

^e^ GS, grazing season, including winter pasture and summer pasture

^f^ T, temperature

^g^, precipitation

^h^ GD, grazing intensity

^i^ T×P, temperature × precipitation.

^j^*df*: degree of freedom

^k^ *P-*values < 0.05.

**Fig 4:**
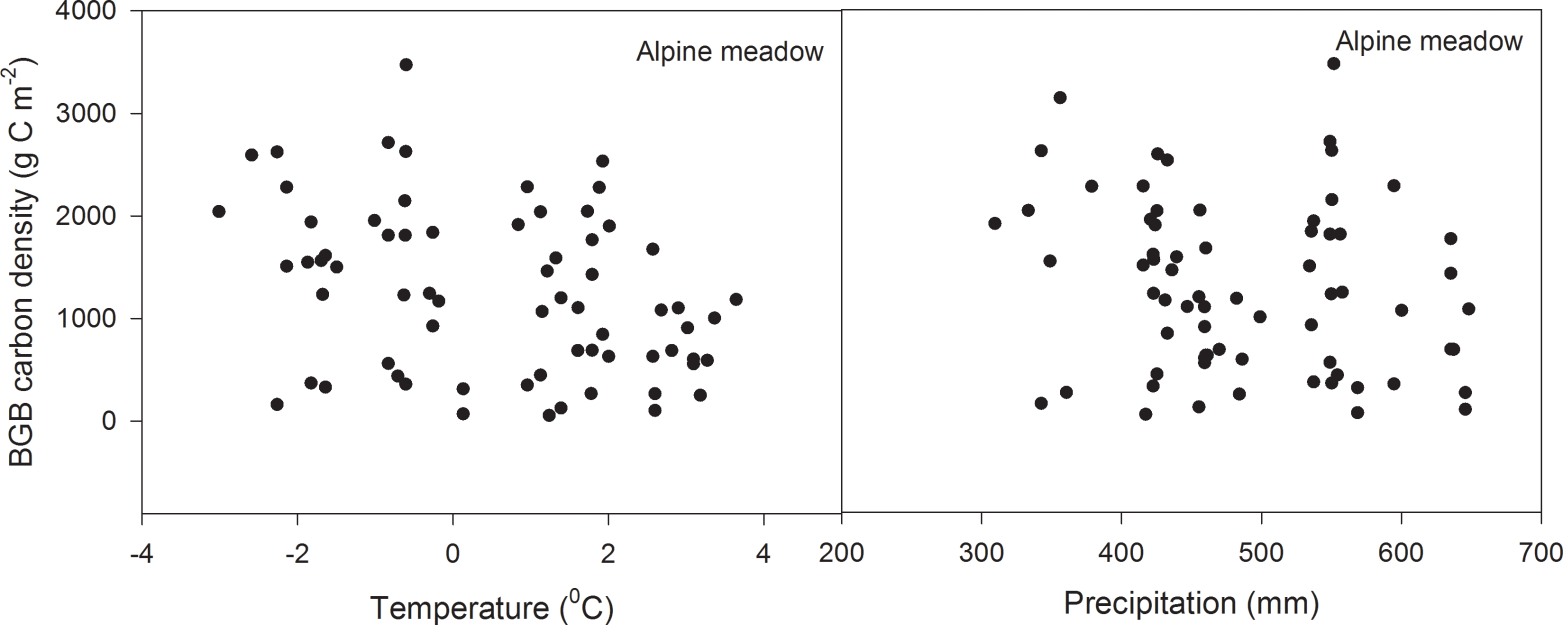
Relationship between belowground biomass (BGB) carbon density and environmental (climate and grazing) factors in alpine grassland. Variation of annual average temperature and annual average precipitation from 1971–2010 in alpine grassland.

Temperature and precipitation interacted to affect BGB carbon density, with the strength of the effect differing between grassland types. Specifically, there was a significant interaction between temperature and precipitation only in alpine meadow (*F* = 4.62, *P* = 0.04). Grazing intensity exerted no impact on BGB carbon density (Table 4, Fig 4).

**Fig 5:**
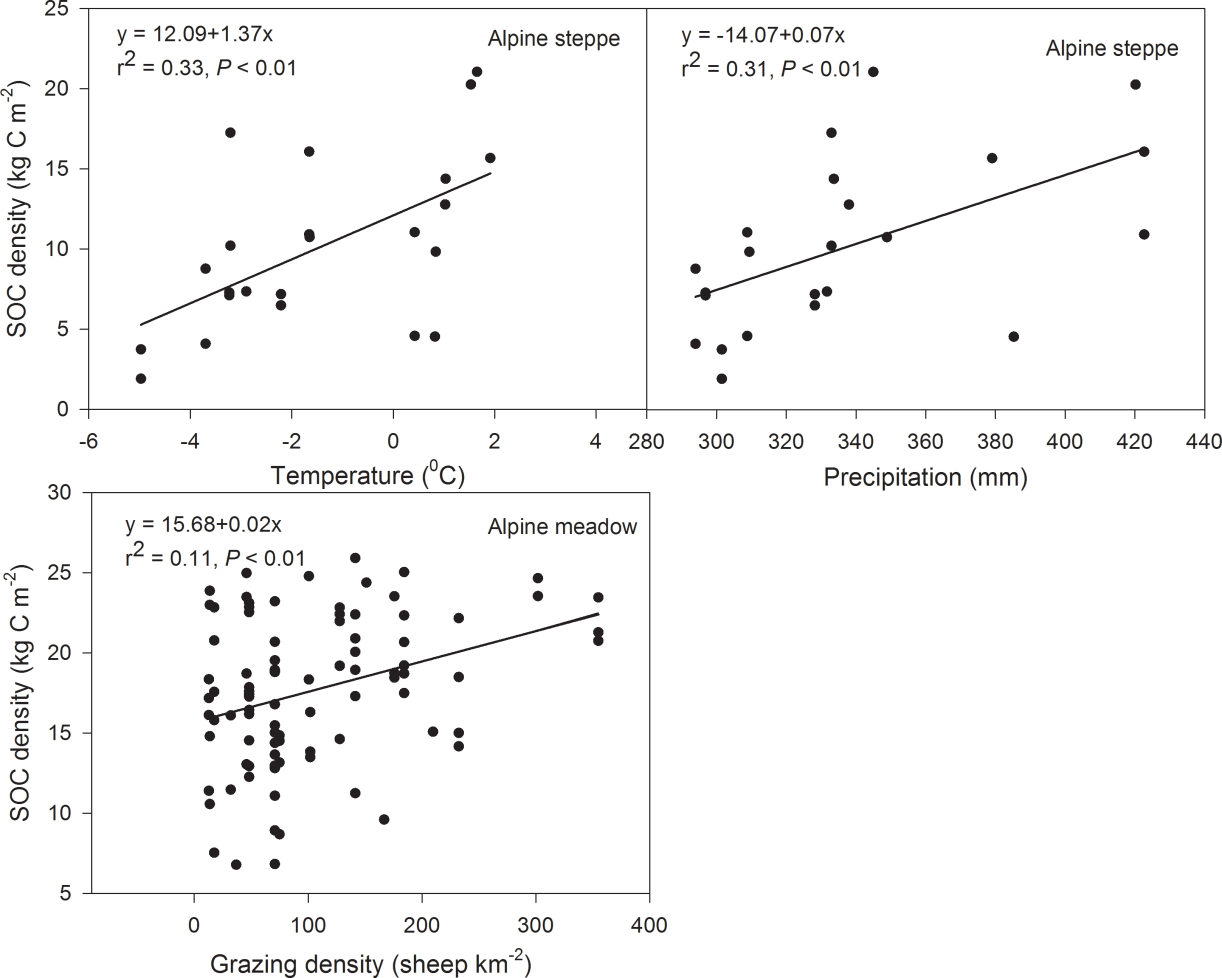
Relationship between soil organic carbon (SOC) density and environmental (climate and grazing) factors in alpine grassland. Variation of annual average temperature and annual average precipitation from 1971–2010 in alpine grassland.

**Fig 6:**
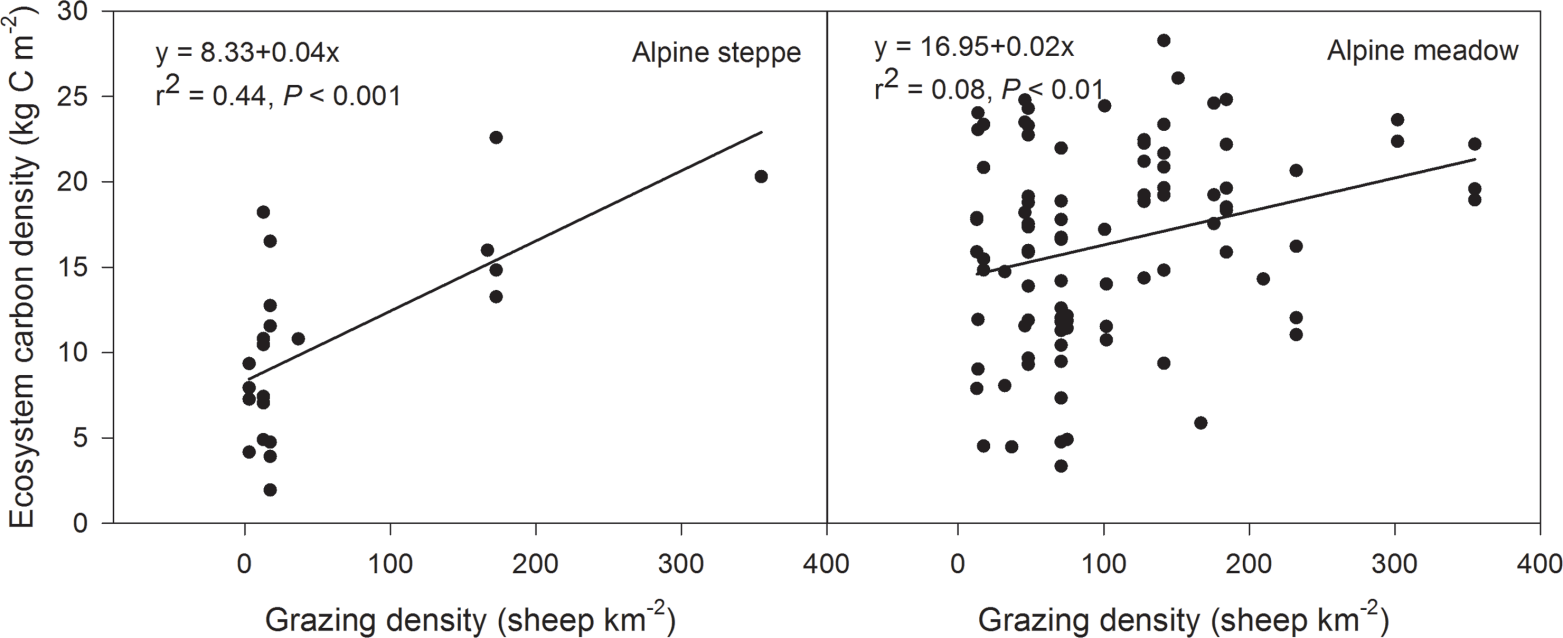
Relationship between ecosystem carbon density and environmental (climate and grazing) factors in alpine grassland. Variation of annual average temperature and annual average precipitation from 1971–2010 in alpine grassland.

Grassland types and grazing intensity exerted significant impacts on SOC density. Temperature and precipitation exerted a strong joint impact on the SOC density in alpine steppe (*F* = 9.04, *P* = 0.01), while grazing intensity alone had a great impact on SOC density in the alpine meadow (Table 4, Fig 5). Both grazing intensity and the type of grassland affected the ecosystem carbon density in the alpine grassland, but grazing intensity was found to be the primary determinant factor of carbon storage (Table 4, Fig 6).

## Discussion

### Effects of climatic factors and grazing intensity on biomass carbon

Quantifying the carbon and biomass budgets is essential for understanding the dynamics of global warming [10]. Rising temperatures are reported to increase vegetation productivity, leading to increasing vegetation carbon storage [26–28]. Further, precipitation also plays a significant role in vegetation dynamics, and consequently any changes in precipitation on the Tibetan Plateau would cause variations in carbon production and storage [20,29,30].

Our results showed that both temperature and precipitation had a positive impact on AGB carbon density in the winter pasture, indicating that temperature and precipitation are limiting factors in the winter pasture of alpine grassland. The lack of livestock disturbance may have allowed unchecked plant growth during the growing season [31], further promoted by the synchronization between peaks of temperature and precipitation. [32], resulting in an increase in AGB carbon density. Notably, the positive correlation between AGB carbon density and temperature in this study contradicts the relationship observed by Yang et al. (2009a) in very dry alpine grassland, suggesting that the enhanced level of precipitation in our sites was a major determining factor [33]. As shown in this study, temperature and precipitation significantly interacted to affect AGB carbon density (Table 4). For BGB carbon density, there was also a significant interaction between temperature and precipitation in alpine grassland, suggesting that BGB is influenced by the combined effect of both temperature and precipitation. SOC increased with increasing temperature and precipitation in alpine steppe.

On the Qinghai-Tibetan Plateau, climate changes have been characterized by rising temperatures and relatively stable humidity [19]. These changes have resulted in increases in AGB carbon storage in the winter pasture and SOC storage in the alpine steppe. On the other hand, these changes may also have stimulated the migration of the plant community, manifested as a shift from alpine meadow to alpine steppe [34]. Given that the total carbon (soil and biomass combined together) in the alpine steppe was less than that in the alpine meadow, plant migration may have been a confounding factor contributing to the discrepancy in carbon densities noted between the two grassland ecosystems. Our results suggest that this migration decreased the ecosystem carbon density (9.35 kg m^-2^).

As discussed above, climate change can significantly influence ecosystem carbon stock in grassland, but human activities can also have a profound effect [35]. Indeed, our results indicated that grazing intensity is the main limiting factor for AGB carbon density in the summer pasture but exerted no impact on BGB carbon density in the alpine grassland. Grazing severely affects plant growth during the growing season and, with consistent exposure to livestock, plants remain unable to undergo normal growth. Interestingly, SOC density in the alpine meadow and ecosystem carbon density in both alpine steppe and alpine meadow increased with an increase in grazing intensity.

Previous studies have reported that grazing reduces the AGB but increases BGB in grazed compared to ungrazed sites [23,36]. In contrast, other studies have found that grazing significantly reduces BGB [37]. Liu et al. (2012) [16] observed that the grazing treatment increased soil carbon storage in desert steppe, which was partly dependent on the grazing history, while in the study of Reeder et al. (2004), it was suggested that long-term grazing decreased the readily mineralizable fraction of soil organic matter [38]. However, Lin et al. (2010) [39] demonstrated that both SOC and BGB carbon increased in response to an initial shift in vegetation carbon to below ground; both quantities then dropped sharply with an increase in grazing pressure. This effect was attributed to changes in the plant community that occurred with grazing disturbance. We believe that the difference between our study and previous studies was likely caused by the difference in sampling time periods. Previous studies showed that both BGB and SOC were affected by long term grazing [37,38]. Our study occurred over data short time scale, which only reflects the grazing intensity over two years. Our results demonstrate that alpine grassland ecosystems with high carbon density are able to support a greater livestock density.

### Ecosystem carbon storage in alpine grassland

Total ecosystem (both biomass and soil) carbon storage in the alpine grassland of the Qinghai Province was estimated at 5.14 Pg, constituting more than 17% of the ecosystem carbon stock in China’s grasslands [35]. In the total alpine ecosystem carbon storage, more than 94% of carbon was stored in the soil and the remaining 6% was stored in vegetation (both above and belowground), indicating that the soil acts as the main carbon store. Soil carbon storage in alpine grassland of Qinghai-Tibetan Plateau has been well researched, yet prior to this study very little was known about the vegetation carbon storage. Estimates of alpine grassland biomass carbon density have varied widely. For example, Ma et al. (2010) [6] reported mean AGB densities of 21.4 and 48.5 g C m^–2^ in the alpine steppe and alpine meadow, respectively, while BGB was 160.3 g C m^–2^ in the alpine steppe and 289.1 g C m^–2^ in the alpine meadow. Fan et al. (2008) [11] found biomass carbon densities ranging from 2.17 kg m^−2^ for alpine meadow to 1.26 kg m^−2^ for alpine steppe. Ni (2002) [12] estimated the median vegetation carbon densities of alpine steppe and alpine meadow as being equivalent (both 1.0 kg C m^-2^). Our estimate of the median biomass carbon density most closely resembled that of Ni (2002), with 0.40 kg C m^-2^ in the alpine steppe and 1.19 kg C m^-2^ in the alpine meadow [12].

Vegetation carbon density is normally based on the field-based observations of biomass [5], and is calculated by multiplying the mean concentration of carbon in the biomass by its respective phytomass [24]. The differences between our estimates and those of previous studies are mainly attributed to two factors: the biomass and the carbon concentration of biomass.

To date, there is very little biomass data available from field-based sample collection [14], particularly for BGB, which is much more difficult to obtain compared to AGB [37]. Previous studies have estimated BGB based on the ratio of BGB to AGB for different vegetation types [8,40]. However, because of seasonal changes in root biomass [41] and the considerable variation in this ratio within the same type of grassland, this method is not accurate. For example, BGB:AGB ratios of steppe and meadow reported by Luo and Li (1998) [42] were 4.33 and 7.92, respectively. Yang et al. (2009b) [43] found that the overall R/S ratio of alpine grasslands was 5.8. Fan et al. (2008) found widely varying BGB:AGB ratios from 40.12 in the alpine steppe to 52.28 in the alpine meadow [11]. Our results indicate a ratio similar to that of Fan et al. (2008), in our case 41.82 in the alpine steppe and 52.96 in the alpine meadow[11].

Conversion coefficients from biomass to carbon ranged from 45% to 55% [44], which can be compared with most previous studies, where 45% has been widely adopted across different grassland types in China [6,11,40]. These values should not be generally applied to all Chinese grassland types, as carbon concentration varies among vegetation types and changes seasonally [45]. Importantly, our large, ground-based dataset indicated that the mean carbon concentration of biomass in alpine grassland is lower than 45%: instead, it should be 35%. This lower value is consistent with that observed in a study by Körner (2003) [46], who claimed that starch becomes less abundant at lower temperatures (except in legumes), which can lead to a lower carbon content in alpine plants. Thus, the conversion factor of 45% may lead to an overestimate of biomass carbon density.

## Conclusions

Biomass estimates on the Tibetan plateau have been widely reported, but owing to the large spatial heterogeneity and lack of direct observations, the accuracy of these estimates has been questioned. In the current study, we addressed some of these issues by using samples from a large number of biomass sites and analyzed these samples directly to obtain representative carbon concentrations. The use of numerous biomass sites also allowed us to assess the impacts of different variables that might affect biomass carbon storage, such as climatic factors and grazing intensity. We found that the mean carbon concentration of biomass was 35% for the alpine grassland, a 10% decrease from previously reported values. We demonstrated that the combined effect of temperature and precipitation significantly impacted on AGB carbon density in winter pasture, BGB carbon density in alpine meadow, and SOC density in alpine steppe. Our results also showed that grazing intensity affected AGB carbon density in summer pasture, SOC density in alpine meadow, and ecosystem carbon density in alpine grassland.

## Supporting Information

S1 Table Description of 115 sites in alpine grassland biomes and carbon distribution across the Qinghai Plateau. Data comprise latitude, longitude, altitude, AGB, BGB, AGB carbon density, BGB carbon density and soil organic carbon density.
(PDF)
