# Supplementary material for: Ecosystem Carbon Storage in Alpine Grassland on the Qinghai Plateau
Source: PLoS One. 2016 Aug 5;11(8):e0160420. doi: 10.1371/journal.pone.0160420 (PMC4975487; doi:10.1371/journal.pone.0160420)
Supplement: S1 Table — Data comprise latitude, longitude, altitude, AGB, BGB, AGB carbon density, BGB carbon density and soil organic carbon density. (PDF) [file pone.0160420.s001.pdf]

	type	logitude	latitude	altitude	agb	agbc	bgb	bgbc
1	alpine st	100.0911	34.02083	4252	61.6425	14.80645	1916.018	549.5883
2	alpine st	99.80972	33.92222	4189	64.154	20.36147	592.1798	228.1963
3	alpine st	99.81056	33.92389	4203	18.145	5.500002	140.6582	61.25759
4	alpine st	99.70278	33.80167	3956	76.2525	28.12427	2361.111	938.1719
5	alpine st	99.70444	33.80722	3974	88.914	20.26088	635.6599	241.0817
6	alpine st	98.6325	35.0325	4382	39.1425	14.00172	171.4437	64.94111
7	alpine st	98.69472	37.53139	3642	191.688	80.58609	2521.6	894.9031
8	alpine st	99.22282	35.3546	4170	87.6384		1103.946	358.9429
9	alpine st	95.49	34.09	4306	113.0172	51.38182	4725.97	1788.305
10	alpine st	95.49	34.09	4306	61.4568	24.93753	1114.137	414.2068
11	alpine st	94.55	34.5	4316	35.0164	12.52085	1446.161	494.6768
12	alpine st	94.54	34.5	4315	35.434	9.539544	415.782	163.402
13	alpine st	93.07	35.15	4622	57.1882	16.27659	528.4206	169.4643
14	alpine st	93.7	35.15	4617	4.167	0.97772	89.26781	35.54919
15	alpine st	92.26	34.12	4546	47.9114	17.52086	1773.56	565.7087
16	alpine st	92.26	34.12	4540	5.76725	1.596441	226.2075	77.95959
17	alpine st	92.18	33.48	4603	35.714	7.873848	1649.903	652.5985
18	alpine st	92.17	33.48	4604	10.92325	3.36271	422.0011	158.2784
19	alpine st	99.53732	37.06738	3233	106.6658	43.67394	1724.232	412.0602
20	alpine st	99.53957	37.0681	3246	179.9804	74.828	1640.773	420.0582
21	alpine st	99.73862	36.75422	3275	111.4466	28.63763	3356.06	1515.702
22	alpine st	101.066	37.06857	3248	187.2384	82.3052	3281.192	1901.768
23	alpine st	100.1278	35.23722	3327	103.912	40.66439		
24	alpine me	101.2756	35.29778	3580	125.538	52.77652		
25	alpine me	101.2714	35.29222	3580	102.1656	39.53095		
26	alpine me	101.3253	35.28306	3635	82.004	26.90654		
27	alpine me	101.4525	35.08222	3725	70.282	29.48249		
28	alpine me	100.8519	30.29111	4401	108.504	41.0354	4529.835	1839.538
29	alpine me	99.88912	35.77003	3528	147.548	50.88476	863.2637	268.3951
30	alpine me	100.9192	35.71389	3370	132.0072	58.8113	3762.752	1501.029
31	alpine me	100.2186	33.47306	4299	98.692	38.28054	953.083	369.8251
32	alpine me	100.2164	33.39111	4288	93.562	35.77437	4624.779	1939.697
33	alpine me	99.38972	33.57306	4077	131.6173	50.4624	1110.253	438.3594
34	alpine me	99.91306	33.57444	4067	160.957	65.90019	4880.166	1811.316
35	alpine me	99.90861	33.56833	4078	111.591	45.12084	7159.015	3140.775
36	alpine me	99.44278	33.6825	4031	185.848	54.48656	176.4862	70.1737
37	alpine me	99.44889	33.67972	4033	191.0256	58.5932	911.2409	313.4579
38	alpine me	99.44917	33.67917	4040	175.008	63.74821	1363.172	560.6037
39	alpine me	99.44889	33.51444	4039	196.312	85.27906	6189.314	2714.981
40	alpine me	99.42611	33.75583	4009	98.728	33.12384	4673.116	1811.55
41	alpine me	99.41889	33.75167	4012	182.2361	74.0238	7349.906	3471.695
42	alpine me	99.16806	34.30444	4219	244.7333	92.37984	6090.101	2627.49
43	alpine me	100.5639	32.77639	4232	147.43	36.96177	1002.831	359.811
44	alpine me	100.5647	32.77667	4207	174.596	65.61359	3329.972	1227.487
45	alpine me	100.5617	32.77556	4257	136.96	45.5996	5939.269	2146.872
46	alpine me	100.4975	33.39028	3912	132.664	36.41669	2755.662	925.6467
47	alpine me	100.4935	33.38611	3900	98.4	29.212	873.7084	266.0423
48	alpine me	100.8519	33.29111	4401	98.216	35.69443	2649.062	1081.04
49	alpine me	100.8519	33.29194	4420	43.2	10.17898	287.3231	102.9764
50	alpine me	100.0617	33.43083	4235	103.1333	24.10891	1445.418	350.5641
51	alpine me	100.9092	33.33472	4111	214.7784	83.06156	3006.104	1430.083
52	alpine me	100.3858	33.26806	4111	172.056	65.47704	5550.425	2283.62
53	alpine me	98.9325	34.94083	4439	46.38667	7.95564	1750.855	689.2663

54	alpine me	98.9325	34.94139	4435	178.288	74.01024	4895.317	1767.662
55	alpine me	98.01111	34.62167	4239	155.152	61.44436	3490.357	1244.304
56	alpine me	98.01556	34.62389	4131	283.07	41.04811	1788.995	686.5408
57	alpine me	98.1225	34.76833	4328	110.752	44.06252	2798.759	1067.312
58	alpine me	98.07222	35.01333	4237	102.43	31.51101	6477.353	2623.394
59	alpine me	98.28972	34.85167	4228	106.112	22.47818	462.1373	160.9774
60	alpine me	98.29389	34.85333	4221	242.48	91.40572	5972.075	2042.427
61	alpine me	98.69361	37.52472	3642	67.784	27.1489	5226.911	1914.53
62	alpine me	99.54385	35.72048	3836	67.8184	19.41271	752.2558	268.111
63	alpine me	97.985	34.5265	4330	239.8144	16.51825	4005.149	1549.135
64	alpine me	97.16845	33.83675	4425	133.8912		3637.739	1589.714
65	alpine me	97.22207	33.87142	4482	148.5416		3742.709	1463.499
66	alpine me	97.095	33.73903	4488	183.5931		3257.519	1104.5
67	alpine me	97.23602	33.35205	4411	163.1383		1706.498	686.4438
68	alpine me	96.61303	32.68267	3947			2663.349	1003.796
69	alpine me	96.66242	32.84703	4282	130.027		1514.361	591.1233
70	alpine me	96.64122	32.89355	4347	27.18286		597.5392	251.734
71	alpine me	96.53	32.51	4001	143.1048		2865.955	1185.565
72	alpine me	96.18	33.18	4391	68.0256	18.68551	361.5888	126.6096
73	alpine me	96.18	33.18	4417	70.42	23.66073	3073.838	1199.644
74	alpine me	96.02	33.34	4303	111.0144		3942.793	1168.216
75	alpine me	95.42	33.49	4232	142.4552		5321.464	1954.966
76	alpine me	95.48	34.06	4219	79.07	29.29678	4187.588	1565.437
77	alpine me	95.51	34.07	4219	125.1304		4142.332	1233.105
78	alpine me	95.51	34.08	4432	70.8888	19.0739	2663.452	1614.385
79	alpine me	95.51	34.08	4428	150.5496		873.5005	329.9813
80	alpine me	100.0139	36.63675	3225	175.9624	76.16633	2000.715	2277.098
81	alpine me	100.7527	37.022	3350	96.2544	40.28707	4851.645	2533.846
82	alpine me	100.7529	37.02277	3336	85.2704	36.10412	2074.435	844.9369
83	alpine me	100.3786	37.90518	3686	107.0467	40.62223	5583.296	2039.981
84	alpine me	100.3756	37.90773	3691	117.5573	36.02901	1254.512	447.1708
85	alpine me	100.2574	38.00585	3985	20.6842	3.827807	177.1231	55.33597
86	alpine me	99.38465	38.59538	3436	110.464	50.03125	4130.551	1510.524
87	alpine me	99.38408	38.59443	3428	116.9064	51.07467	5698.13	2280.359
88	alpine me	99.53638	38.46792	3305	137.812	55.27258	6493.336	2592.509
89	alpine me	101.1773	37.66925	3335	67.356	29.27034	4801.486	2045.669
90	alpine me	101.2978	37.62035	3527	82.26667	34.34117	1645.362	630.0638
91	alpine me	100.8983	37.96528	3200	331.0816	144.2732	1794.276	604.3959
92	alpine me	101.0489	37.87167	3189	218.7128	89.59963	1624.027	557.0719
93	alpine me	101.3158	37.61306	3278	159.1208	62.86868	2968.728	1101.698
94	alpine me	101.3064	37.61	3239	130.3368	51.81223	4112.851	1675.655
95	alpine me	101.2578	37.70222	3241	129.7216	55.7512	1724.235	630.2663
96	alpine me	101.1822	37.68167	3204	355.3808	155.2854	2604.462	908.3276
97	alpine me	101.7356	34.65167	3593	130.57	41.45507		
98	alpine me	101.7489	34.66444	3596	243.6816	105.097		
99	alpine me	100.8672	34.97417	3800	109.6128	39.06028		
100	alpine me	100.8658	34.97194	3802	132.922	57.66733		
101	alpine me	100.8647	34.97028	3786	75.2016	30.06517		
102	alpine me	100.5719	34.34	4084	137.8904	42.08449		
103	alpine me	100.5719	34.33972	4081	71.7512	27.33315		
104	alpine me	100.4964	34.3525	3957	124.0232	45.62918		
105	alpine me	100.4925	34.36583	3955	129.5952	55.70699		
106	alpine me	100.4922	34.35806	3948	239.1752	99.40376		
107	alpine me	100.2808	34.42111	3768	77.2176	29.28586		

108	alpine me	100.4058	34.47833	4328	216.0776	90.00468
109	alpine me	100.2728	34.39361	3820	82.556	33.92316
110	alpine me	100.1358	34.15056	4089	140.3347	39.54066
111	alpine me	100.1358	34.15528	4091	131.8056	
112	alpine me	100.6094	34.32972	4151	88.5368	27.71692
113	alpine me	100.6075	34.32833	4145	77.032	22.43952
114	alpine me	100.0853	34.08194	4212	98.1736	35.29649
115	alpine me	100.085	34.06833	4292	35.276	12.585

soc
6.484001
7.178189
7.349011
17.25103
10.19863
10.73658
9.828773
4.536492
10.90402
16.07289
11.04238
4.577708
3.733823
1.909889
8.770097
4.089461
7.276436
7.1098
14.37356
12.76666
21.04537
23.51554
20.25689
22.1599
14.16764
15.00411
18.48816
12.96189
15.67027
15.80023
8.68362
13.16134
12.26278
16.43247
16.43247
12.93693
14.53498
17.60088
17.85531
15.07702
17.41507
22.5287
16.17762
23.11426
17.26669
6.830641
19.52291
13.49201
13.83998
16.30169
19.1919
14.62349
21.97125

19.17833
22.41385
22.81697
22.84124
17.17202
11.39485
16.11537
6.788638
9.606937
18.34411
13.04814
18.70695
23.47328
24.96827
18.33443
24.77735
11.4682
16.09545
14.79767
22.9855
23.86127
10.57131
20.76621
7.538972
22.82998
17.56433
24.36943
18.45432
18.74068
18.9304
20.89118
17.29204
11.24947
20.05305
25.90733
22.39212
22.38852
17.48817
25.03215
22.32507
18.70913
20.6661
19.20717
24.6492
23.53618
21.27779
23.44659
20.74843
20.68063
11.08725
16.78577
18.79912
23.20801
15.4747

12.809
13.65386
8.926097
15.02563
14.38211
18.93104
14.85002
14.51011
